# Photo-products of retinal pigment epithelial bisretinoids react with cellular thiols

**Published:** 2011-07-08

**Authors:** Kee Dong Yoon, Kazunori Yamamoto, Jilin Zhou, Janet R. Sparrow

**Affiliations:** 1 Department of Ophthalmology, Columbia University, New York, NY; 2 Department of Pathology and Cell Biology, Columbia University, New York, NY

## Abstract

**Purpose:**

Bisretinoids such as A2E that accumulate as components of the lipofuscin of retinal pigment epithelial cells are implicated in some retinal disease processes. These compounds undergo light-induced oxidation and cleavage with the latter releasing of a mixture of aldehyde-bearing fragments, including dicarbonyl methylglyoxal. We tested for the reactivity of photooxidation and photodegradation products of A2E with thiol-containing glutathione (GSH).

**Methods:**

In cell-free assays, we measured the ability of photooxo-A2E to competitively inhibit the GSH-mediated reduction of the thiol reagent 5,5′-dithiobis-(2-nitrobenzoic acid). Cellular GSH was assayed colorimetrically. Products of GSH reduction and GSH-adducts were detected by electrospray ionization mass spectrometry (ESI-MS) and GSH and oxidized GSH (glutathione disulfide [GSSG]) were quantified from chromatographic peak areas.

**Results:**

We found that GSH can donate hydrogen atoms to, and form conjugates with, photooxidized forms of the bisretinoid A2E and with its photocleavage products. Reaction with non-photooxidized A2E was not observed. Chemical reduction by GSH involved the donation of a hydrogen atom from each of two GSHs. The ratio of GSH consumed to GSSG formed was consistent with GSH being used for both reduction and adduct formation. With the aid of synthesized standards, methylglyoxal-GSH adducts were identified within mixtures of GSH and photooxidized A2E; the adducts formed noncatalytically and by glutathione-S-transferase mediation.

**Conclusions:**

Reduction and adduct formation by GSH likely limits the reactivity of bisretinoid photoproducts and may aid their elimination from the cells. These findings are significant to forms of macular degeneration associated with bisretinoid formation and maculopathy stemming from GSH synthase deficiency.

## Introduction

The tripeptide glutathione (l-γ-glutamyl-l-cysteinyl-glycine; GSH^2^) protects cells against electrophiles such as aldehydes and ketones, and against reactive oxygen species; GSH does so by donating a hydrogen atom (H^+^ + e^-^) from the thiol (-SH) group of its cysteine residue. With the loss of an electron, GSH is converted to a radical (GS•) that subsequently reacts with a second oxidized GSH molecule, thereby creating glutathione disulfide (GSSG) [1]. In addition, GSH can form conjugates with small molecules in reactions that occur nonenzymatically or that are catalyzed by glutathione-S-transferase enzymes (GST); the latter enzyme-catalyzed reaction proceeds at a rate that is many times faster [1]. The formation of GSH conjugates is important for the detoxification of many compounds.

GSH is present in cells in millimolar concentrations [1]. It is synthesized in the cytosol by two ATP (ATP)-requiring processes. First, the dipeptide gamma-glutamylcysteine is synthesized from l-glutamate and cysteine in a rate-limiting step catalyzed by glutamate cysteine ligase; then glycine is added via the enzyme GSH synthetase. The synthesis of GSH is also controlled by cysteine availability [2].

All cells must contend with reactive oxygen species (superoxide anion, hydrogen peroxide) that are produced within mitochondria as a result of the incomplete reduction of oxygen, the final acceptor in the electron transport chain [1]. In addition, however, retinal pigment epithelium (RPE) cells of the eye are confronted with an unusual source of oxidative insult—the photooxidative processes that originate within the bisretinoid compounds that comprise the lipofuscin of these cells. The bisretinoids of the RPE constitute a complex mixture that originates in photoreceptor cell outer segments from reactions mediated by all-*trans*-retinal, the retinoid that forms upon photoisomerization of the visual pigment chromophore 11-*cis*-retinal [3]. Deposition of the bisretinoids in RPE cells occurs during the normal process of outer segment shedding and phagocytosis. To date, numerous components of RPE lipofuscin have been structurally characterized, including A2E and its isomers [4-7]; A2-dihydropyridine-ethanolamine (A2-DHP-E) [8]; and compounds of the all-*trans*-retinal dimer series: all-*trans*-retinal dimer, all-*trans*-retinal dimer-phosphatidylethanolamine (all-*trans*-retinal dimer-PE) and all-*trans*-retinal dimer-ethanolamine (all-*trans*-retinal dimer-E) [9]. A structural feature common to all of these di-retinal pigments is dual conjugation systems that confer absorbances in both the ultraviolet and visible light spectrum (A2E: λ_max_, 335, 439 nm; A2-DHP-PE: λ_max_ 333, 490 nm; all-*trans*-retinal dimer: λ_max_, 290, 432 nm). In the case of all-*trans*-retinal dimer-PE and all-*trans*-retinal dimer-E, an additional red-shift to 510 nm is associated with protonation of the Schiff base nitrogen [9]. Using A2E as a bisretinoid model, we have shown through a variety of approaches that this pigment serves as both a photogenerator and quencher of singlet oxygen with photooxidation-induced cleavage of the molecule occurring at sites of molecular singlet oxygen cycloaddition [10-12]. The mixture of aldehyde-bearing products released upon photodegradation of A2E includes methylglyoxal (MG), a low molecular weight reactive dicarbonyl that is an agent responsible for advanced glycation endproduct (AGE) modification of proteins [13]. It is significant that AGE-modified proteins are detected in deposits (drusen) [14-16] that accumulate below RPE cells in vivo; drusen have been linked to age-related macular degeneration pathogenesis [17]. These findings suggest a possible association between RPE lipofuscin photooxidation/photodegradation and drusen formation.

Here, we sought to determine whether GSH could neutralize photodegradation products of A2E by donating reducing equivalents and/or by forming conjugates.

## Methods

### Cell culture

A human adult retinal pigment epithelial cell line (ARPE-19; American Type Culture Collection, Manassas, VA) lacking endogenous A2E was grown to confluence in Dulbecco’s Modified Eagle Medium (Invitrogen, Carlsbad, CA), 5% fetal calf serum (Invitrogen, Carlsbad, CA), 2 mM glutamine (Invitrogen) and gentamicin sulfate [18]. Subsequently, synthesized A2E (10 μM in culture medium) was introduced to the cultures for accumulation in the lysosomal compartments of the cells [18]. Cells were exposed to 430 nm (±20 nm) light (1.38 mW/cm^2^; 20 min).

### Synthesis of compounds

A2E was synthesized by incubating all-trans-retinal with ethanolamine as published [6] and all-trans-retinal dimer was synthesized by treating all-trans-retinal with sodium hydride as previously described [19]. To prepare photooxidized A2E (photooxo-A2E), stock A2E in DMSO (DMSO) was diluted in 200 µl PBS to obtain 50, 100, and 200 µM concentrations. Samples were irradiated (430±20 nm) for 3 (50 µM) 5 (100 µM) and 15 (200 µM) min. In other experiments, A2E and all-*trans*-retinal dimers (200 µM) were irradiated for 7 min. Peroxy-A2E was synthesized by reaction with 1,4-dimethylnaphthalene endoperoxide, as previously described [20].

### Colorimetric assay of reactivity between between glutathione and oxidized-A2E

Reactivity of oxo-A2E with GSH was assayed by measuring residual GSH available for reduction of 5,5′-dithiobis-(2-nitrobenzoic acid; DTNB) to the yellow-colored product 2-nitro-5-thiobenzoic acid (TNB). Accordingly, GSH (100 microM; ApoGSH Glutathione Colorimetric Detection Kit; BioVision Research Products, Mountain View, CA) in buffer containing 1% sulfosalicylic acid was incubated with and without 50, 100, and 200 microM photooxo-A2E, peroxy-A2E, and/or photooxo-all-*trans*-retinal dimer for 3 h at 37 °C. Where indicated, the reaction mixture included GSH reductase and nicotinamide adenine dinucleotide phosphate-oxidase (NADPH; BioVision Research Products; reagents of ApoGSH kit). Subsequently, DTNB (60 μM; BioVision Research Products) was added and after 10 min, absorbance at 405 nm was read in an MRX Revelation microplate reader (Dynex Technologies, Inc., Chantilly, VA). Background absorbance (photooxo-A2E-only samples) was subtracted and normalization to GSH-only values was performed as indicated. In all experiments, data were collected in duplicate.

### Assaying cellular glutathione

Supernatants from cell lysates containing 1% sulfosalicylic acid were submitted to GSH colorimetric assay in the presence of GSH reductase, NADPH, and DTNB, as per kit instructions (BioVision Research Products). Absorbance was read at 405 nm, GSH concentration was determined by reference to a GSH calibration curve, and protein concentrations were measured by Bio-Rad protein assay kit (Bio-Rad, Hercules, CA).

### High-performance liquid chromatography and ultra-performance liquid chromatography–mass spectrometry analysis

Mixtures of A2E (200 μM; 180 μl) and GSH (0.5–20 milliM) were irradiated at 430±20 nm for 20 min. When indicated, A2E (200 μM) in water was irradiated then extracted with chloroform and evaporated under argon gas. Pooled samples were redissolved in PBS, GSH (20 mM) and GST (1 μg/μl) were added, and the mixture was incubated for 1 h at room temperature. In control samples, water was substituted for GSH. In other experiments, A2E (200 μM) and all-*trans*-retinal dimer (200 μM) were irradiated at 430 nm for 0, 15, 30, 60, 120, and 240 s. Additionally, MG (50 mM; Sigma-Aldrich, St. Louis, MO), GSH (100 mM), and GST (1 μg/μl) were incubated for 1 h before ultra-performance liquid chromatography (UPLC)-MS analysis.

For high-performance liquid chromatography, compound elution was achieved using an Alliance system (Waters Corp Milford, MA) with a Delta Pak^®^ C4 (5 µm, 3.9×150 mm; Waters) or an Atlantis^®^ dC18 (3 µm, 4.6×150 mm; Waters) column. Chromatographic solvents were obtained from Sigma-Aldrich or Fisher Scientific (Pittsburgh, PA). The mobile phase for the C4 column was a gradient of acetonitrile in water with 0.1% trifluoroacetic acid: 0–5 min, 75% acetonitrile, flow rate, 0.8 ml/min; 5–30 min, 75%–100% acetonitrile, flow rate, 0.8 ml/min; 30–35 min, 100% acetonitrile, flow rate, 0.8–1.2 ml/min; 35–50 min, 100% acetonitrile, flow rate, 1.2 ml/min. With the dC18 column, a gradient of acetonitrile in water with 0.1% trifluoroacetic acid was used: 75%–90% acetonitrile (0–30 min); 90%–100% acetonitrile (30–40 min); and 100% acetonitrile (40–100 min) with a flow rate of 0.5 ml/min. Absorbance (Waters 2996 Photodiode Array) and fluorescence (Waters 2475 Multi λ Fluorescence Detector; 18 nm bandwidth) were detected at the wavelengths indicated.

UPLC-MS analysis was performed on a Waters Acquity UPLC system (Waters Corp, Milford, MA) that was coupled online with a Waters SQD single quadrupole mass spectrometer and both PDA eλ and fluorescence (FLR; Waters) detectors. The mass spectrometer was equipped with electrospray ion multimode ionization and ion trap analyzer operating in full-scan mode from a mass to charge ratio (*m/z)* of 300–1200. For GSH elution, an Atlantis^®^ dC18 column (3.0 µm, 150 × 2.1 mm I.D.) was used for the stationary phase with an isocratic mobile phase (2%) of a 1:1 mixture of acetonitrile and methanol (0–5 min) with 0.1% formic acid, a flow rate of 0.5 ml/min, and injection volume of 10 μl. For GSSG elution, a Luna C18 column (3.0 µm, 50×2.0 mm I.D.) was used for the stationary phase and 0.1% formic acid (A) and a 1:1 mixture of acetonitrile and methanol with 0.1% formic acid (B) were used (0.0–0.5 min, 0% B; 0.5–0.6 min, 0%–97% B; 0.6–2.0 min, 97% B) for the gradient elution. The flow rate was 0.6 ml/min with an injection volume of 10 μl. GSH and GSSG were quantified by comparison to external calibration curves obtained by analyzing a series of sequentially diluted solutions of GSH (0.05, 0.25, 0.5, and 1.0 milliM) and GSSG (3.125, 12.5, 50, and 200 μM).

For A2E elution, an Xbridge^®^ C18 column (2.5 µm, 3.0×50 mm I.D.) was used with a linear gradient (65%–90%) of a 1:1 mixture of acetonitrile and methanol (0–8 min) with 0.1% formic acid, a flow rate of 0.5 ml/min, and an injection volume of 5.0 microL. For all-*trans*-retinal dimer elution, an Xbridge^®^ C8 column (2.5 µm, 50×3.0 mm I.D.) was used with a gradient of formic acid (0.1%; A) and acetonitrile/methanol (1:1) with 0.1% formic acid (B; 0.0–4.5 min, 85% B; 4.5–5.0 min, 85%–100% B; 5.0–10 min, 100% B). The flow rate was 0.6 μl/min with an injection volume of 5.0 μl.

### Statistical analysis

One-way ANOVA and Newman Keul Multiple Comparison test were applied using statistical software (Prism, GraphPad Software).

## Results

### Reaction of photooxidized-A2E with glutathione

**Figure 1:**
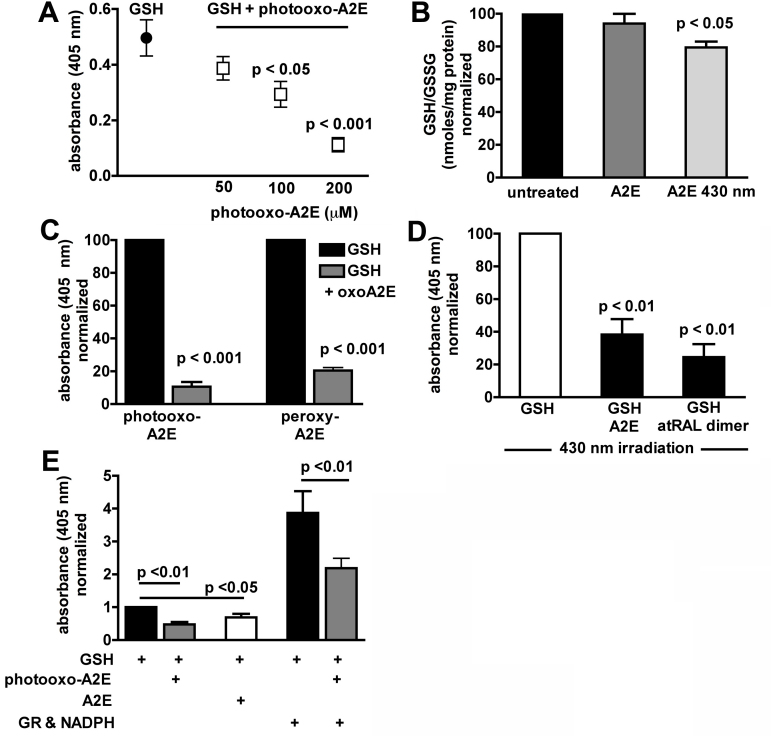
Reaction of photooxidized bisretinoid with glutathione and glyceraldehyde-3-phosphate dehydrogenase. Data are mean±standard error of mean (SEM) of 3–5 experiments. **A**: Incubation of glutathione (GSH) with photooxidized-A2E (photooxo-A2E) results in a concentration-dependent decrease in GSH-mediated reduction of 5,5′-dithiobis-2-nitrobenzoic acid (DTNB) to 2-nitro-5-thiobenzoic acid (TNB). Background absorbance (DPBS) and absorbance values for photooxo-A2E samples alone were subtracted. **B**: Total glutathione (GSH/GSSG) in ARPE-19 cells that had accumulated A2E and were exposed to 430 nm light. Total glutathione was measured in a DTNB-based assay in the presence of glutathione reductase and nicotinamide adenine dinucleotide phosphate (NADPH), and values were normalized to untreated controls. **C**: Incubation of GSH (650 µM) with photooxo-A2E and peroxy-A2E (100 µM) diminishes the ability of GSH to subsequently reduce DTNB to TNB. DPBS and absorbance values for photooxo-A2E and peroxy-A2E alone were subtracted and data were normalized to GSH control. **D**: A2E and all-*trans*-retinal dimer were irradiated in the presence of GSH, after which DTNB was added. The background was subtracted and values normalized to the GSH control. **E**: Prior incubation of GSH with photooxo-A2E decreases the ability of GSH to reduce DTNB even in the presence of GSH reductase (GR) and NADPH. DPBS and absorbance values for photooxo-A2E samples alone were subtracted and data were normalized to GSH only.

To examine for reaction between photooxo-A2E and GSH, we began by measuring the ability of photooxo-A2E to competitively inhibit GSH-mediated reduction of the thiol reagent DTNB, a reaction that produces the yellow-colored product TNB. By colorimetric assay in the absence of GSH reductase, the rate of TNB generated is directly proportional to the amount of GSH present [21]. Accordingly, in samples of GSH that had been incubated with photooxo-A2E, we observed a concentration-dependent decrease in absorbance at 405 nm, indicative of a decline in TNB formation due to diminished availability of GSH (Figure 1A). In keeping with the latter observation, we also measured a 20% decrease in intracellular GSH when ARPE-19 cells that had accumulated A2E were irradiated at 430 nm (Figure 1B). To probe for evidence that GSH could react with a specific form of oxidized A2E, we synthesized peroxy-A2E, an oxidized species that carries an endoperoxide due to the cycloaddition of singlet oxygen. Incubation with this synthesized form of oxidized A2E resulted in similar decreases in TNB formation (Figure 1C).

**Figure 2:**
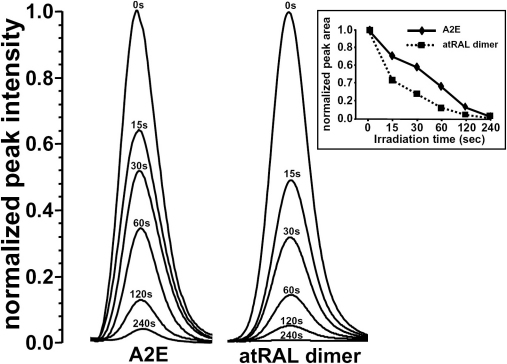
Photooxidation of A2E and all-trans-retinal dimer (at RAL dimer)monitored by UPLC with photodiode array detection. A2E and atRAL dimer were irradiated at 430 nm for indicates times. Absorbance peaks were normalized to intensity at 0 time. Inset; normalized absorbance peak area as a function of time. Decreases in peak height and area reflect photooxidation-associated consumption of the compound.

Photooxidation of the bisretinoid all-*trans*-retinal dimer by exposure to 430 nm light also reduced GSH-associated DTNB reduction (Figure 1D); here, the inhibition associated with the photooxidized all-trans-retinal dimer was modestly greater than that mediated by photooxo-A2E (photooxo-A2E, 50% decrease; photooxo-all-*trans*-retinal dimer, 59%). The latter difference reflected a greater capacity for all-*trans*-retinal dimer to photooxidize, since when we compared the tendencies of A2E and all-*trans*-retinal dimer to undergo photooxidation as measured by loss of corresponding bisretinoid, consumption of the all-*trans*-retinal dimer occurred at a faster rate (Figure 2).

The disulfide dimer GSSG that forms upon oxidation of GSH can be recycled back to GSH by GSH reductase using NADPH as a cofactor. The regenerated GSH subsequently reacts with DTNB to produce more TNB. As expected, therefore, inclusion of glutathionine reductase and NADPH in the reaction mixture amplified the production of TNB (Figure 1E). Nevertheless, with the addition of photooxo-A2E, the 405 nm absorbance due to TNB production was still diminished.

### Ultraperformance liquid chromatography-mass spectrometry analysis

**Figure 3:**
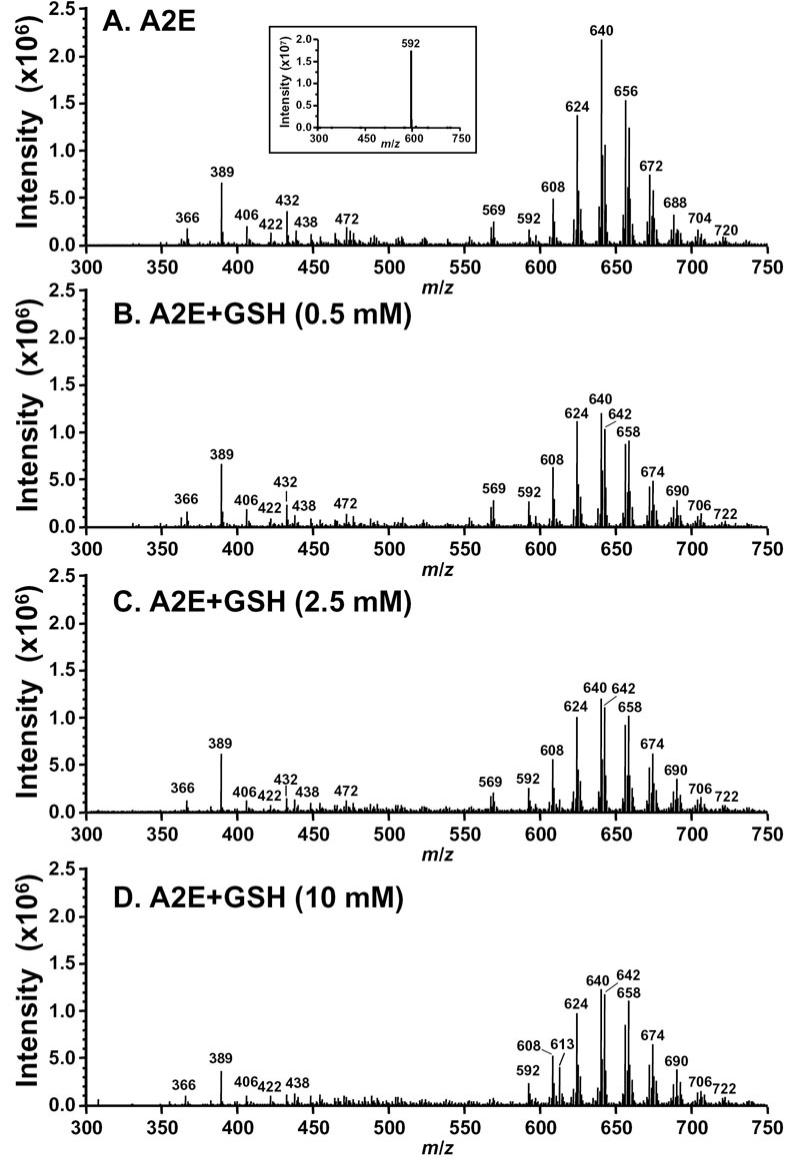
Electrospray ionization (ESI) mass spectra of samples of A2E irradiated in the absence and presence of glutathione (GSH). Mixtures of A2E and GSH at indicated concentrations were irradiated at 430 nm for 20 min following which the sample was analyzed by ultraperformance liquid chromatography–mass spectrometry (UPLC/MS). The mass to charge ratio (*m/z*) of glutathione disulfide (GSSG) is 613.

**Figure 4:**
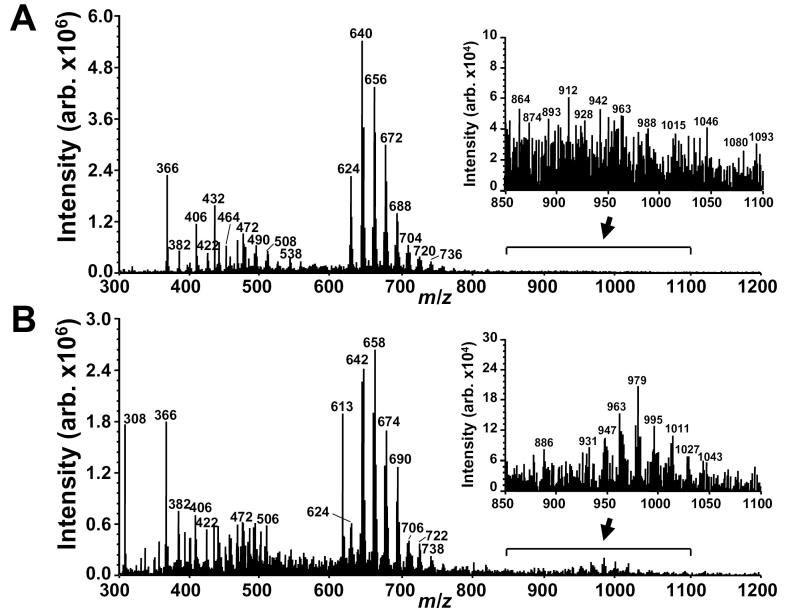
Electrospray ionization (ESI) mass spectral analysis of A2E irradiated (430 nm, 20 min) in the absence (**A**) and presence (**B**) of glutathione (GSH). A2E, m.w. 592, 200 μM; GSH, 10 mM. **A**: The series of peaks at 592 <*m/z* <736 reflect A2E photooxidation products (592 + n*16, n=1, 2…9). Lower mass peaks (<*m/z* 592) correspond to A2E photocleavage products. **B**: The peaks at 592 < *m/z* <738 exhibit *m/z*+2 relative to A2E photooxidation products in A. The series of peaks at *m/z* 931, 947, 963, 979, 995, 1011, 1027, and 1043 are photooxoA2E-GSH adducts (mechanisms of formation are proposed in Figure 6). Insets in **A** and **B**: expanded view in range of *m/z* 850–1100. Note that the y-axis scale is magnified in B to facilitate resolution.

For further evidence of activity between GSH and photooxidation products of A2E, we irradiated A2E at 430 nm in the absence and presence of GSH (307 Da) and analyzed by MS using an ESI source operating in positive ion mode. The ESI spectra revealed a considerable reduction of the *m/z* (mass-to-charge ratio) 592 peak attributable to A2E (Figure 3A, inset) together with a display of molecular ion peaks (*m/z* 608, 624, 640, 656, 672, 688, 704, 720) known from our previous work to reflect A2E photooxidation products [11] (Figure 3A). We have previously demonstrated by nuclear magnetic resonance (NMR), that the oxygen-containing moieties generated within photooxidized-A2E include furans and endoperoxides [11]. The addition of GSH to the irradiated sample changed the MS pattern in the region of the spectrum occupied by photooxidized species of A2E (*m/z* 600–750; Figure 3B-D). Specifically, in this region some *m/z* peaks became coupled to additional *m/z* signals exhibiting a mass shift of +2 Da (e.g., *m/z* 640/642, 656/658, 672/674, 688/690, 704/706, 720/722) (Figure 3B-D; Figure 4B; *m/z* 736/738 is also detected in Figure 4B). This change is consistent with donation of hydrogen atoms from the thiol groups of each of two GSH molecules. Interestingly, these adducts preferentially formed with photooxidized forms of A2E to which three or more oxygen atoms had been added at former sites of carbon-carbon double bonds (*m/z* ≥640). Further evidence of GSH oxidation was provided by detection of the *m/z* 613 peak attributable to GSSG (Figure 3D). A mixture of lower molecular weight *m/z* signals (*m/z* 350–500) was also present in the sample of A2E irradiated in the absence of GSH, some of which (*m/z* 422, 432, 438; Figure 4A-D), with our previous structural characterization, were revealed to be photooxidation-induced cleavage products of A2E [12]. It was noted that at the highest GSH concentration, at least one of these peaks (*m/z* 432) was absent, a change indicative of GSH adduct formation (Figure 3A-D).

Extending the *m/z* range (*m/z* 300–1200) in an additional experiment provided further evidence of the formation of GSH-conjugates (Figure 4). Here, irradiation of A2E was accompanied by photooxidized species of A2E typically observed in the *m/z* region 600–750 while A2E photocleavage products resided in the region of *m/z* 350–500. Significantly, with the addition of GSH to A2E before irradiation, two changes in the MS pattern were observed. First, the *m/z* signals corresponding to A2E photooxidation products (600–750 *m/z*) exhibited a mass shift of +2 Da, which was indicative of hydrogen transfer from GSH. Second, a series of higher molecular weight peaks (*m/z* 931, 947, 963, 979, 995, 1011, 1027, 1043) appeared that were indicative of GSH-adduct formation involving nonenzymatic nucleophilic attack of photooxidized forms of A2E by GSH (592+(n*16)+GSH, n=2, 3, 4…). Interestingly, these adducts preferentially formed with photooxidized forms of A2E to which two or more oxygen atoms have been added at former sites of carbon-carbon double bonds. Evidence of hydrogen atom donation by GSH was also provided by the appearance of the *m/z* 613 peak attributable to GSSG (Figure 4B).

**Figure 5:**
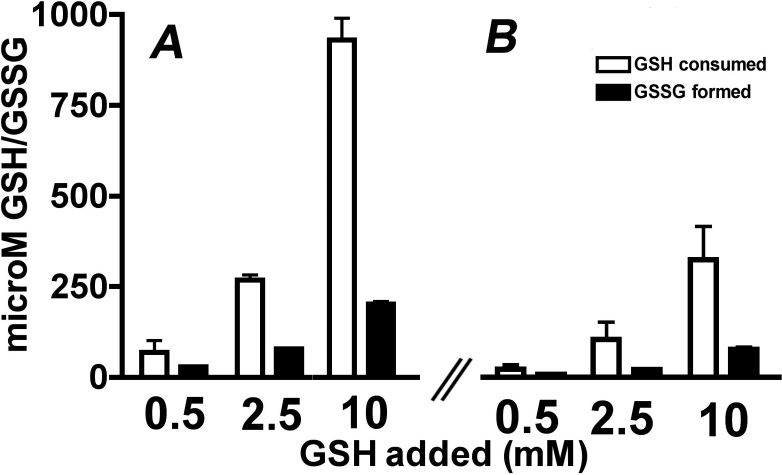
Ultraperformance liquid chromatographic quantification of glutathione (GSH) consumed and glutathione disulfide (GSSG) formed when irradiated A2E is incubated with GSH. **A**: A2E (200 μM) was irradiated (430 nm, 20 min) in the presence of GSH at the indicated concentrations. **B**: A2E (200 μM) was irradiated (430 nm, 20 min) and then incubated with GSH.

Quantification of GSH and GSSG from chromatographic peak areas revealed that the ratio of GSH consumed/GSSG formed was higher when GSH was present during A2E irradiation (Figure 5 A) as opposed to being added after A2E irradiation (Figure 5 B). Although one equivalent of GSSG forms upon hydrogen atom donation from two equivalents of GSH, the ratio of GSH consumed/GSSG formed was greater than 2:1, consistent with GSH being used both for donation of a hydrogen to photooxidized A2E (GSSG is generated) and for the formation of GSH adducts (GSSG is not generated).

**Figure 6:**
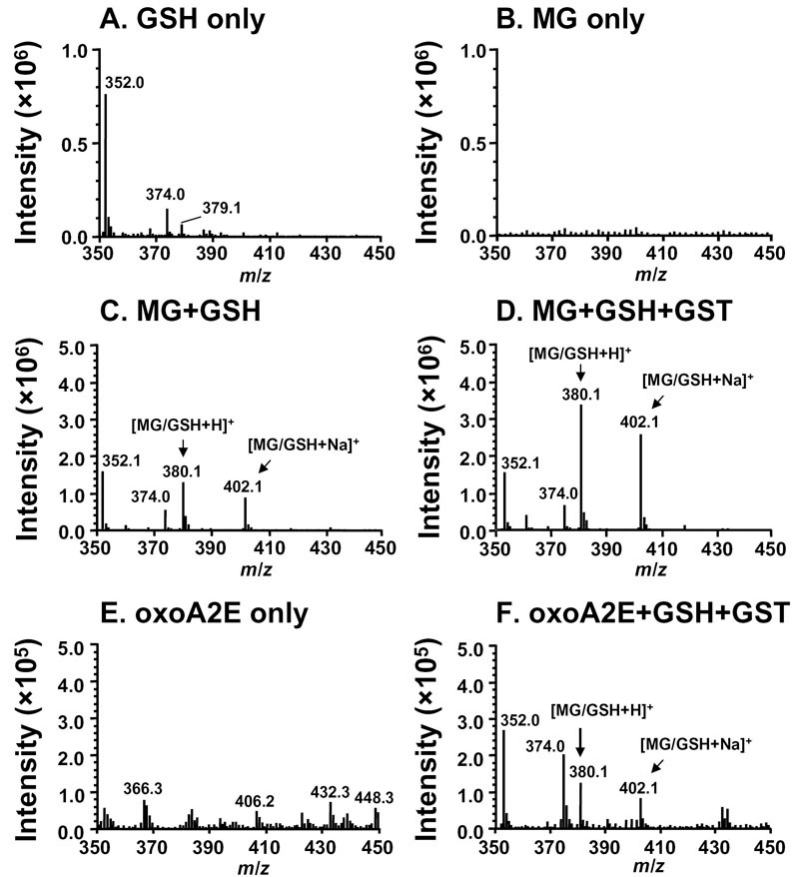
Electrospray ionization (ESI) mass spectrometric detection of methylglyoxal (MG)-glutathione (GSH) adducts. **A**: GSH incubated alone. **B**: MG incubated alone. **C**: Incubation of MG (50 μM)+GSH (100 μM) in the absence of glutathione-S-transferase (GST). **D**: Incubation of MG (50 μM)+GSH (100 μM) in the presence of glutathione-S-transferase (MG+GSH^+^GST). **E**: Incubation of photooxidized A2E (oxoA2E) only. **F**: Incubation of GSH with photooxidized A2E and GST (oxoA2E+GSH^+^GST). The *m/z* 380.1 signal is attributable to the protonated ion [MG/GSH^+^H]+. The molecular ion species m/z 402.1 is attributable to [MG/GSH^+^Na]+ under positive ion mode.

We previously showed that one of the molecular fragments (72 Da) released upon photocleavage of A2E is the toxic dicarbonyl MG [12]. To determine whether GSH forms a conjugate with MG generated by A2E photocleavage, we first reacted GSH with commercially available MG in the presence and absence of GSH transferase (GST), analyzed by ESI-MS, and observed the expected GSH/MG adduct at *m/z* 380 (m.w. 307 + 72) (Figure 6C,D); the latter adduct was not present in samples of GSH (Figure 6A) or MG (Figure 6B) alone. The GSH-MG adduct later presented as a molecular ion [M+H]^+^ at *m/z* 380 when protonated in positive ion MS ([GSH/MG+H]^+^; 307+72+1) and at *m/z* 402 when sodiated ([GSH/MG+Na]^+^; [379+23]^+^ (Figure 6C,D). Sodiation of GSH adducts under MS analysis has been described [22]. The presumed GSH-MG adduct formed nonenzymatically in the absence of GST but with the addition of GST, the yield was greater. To test for the facile reaction of GSH with MG released upon A2E photocleavage, samples of irradiated A2E were extracted with chloroform, dried, reconstituted in PBS, and then incubated with GSH in the presence of GST. Again, the *m/z* 380 and *m/z* 402 signal was observed (Figure 6E,F).

## Discussion

The bisretinoids of RPE lipofuscin are considered to lead to retinal degeneration in early onset blinding disorders associated with mutations in the genes encoding ATP-binding cassette sub-family A member 4 (ABCA4) [23,24], and have been implicated in retinal disease caused by mutations in elongation of very long chain fatty acids-4 (*ELOVL4*) [25]. The deposition of these pigments may also contribute to the etiology of age-related macular degeneration [3]. The photoreactivity of these pigments in response to excitation by wavelengths in the visible spectrum likely contributes to the adverse effects of their accumulation. Specifically, photoexcitation of bisretinoids such as A2E and all-*trans*-retinal dimer leads to the production of reactive forms of oxygen, particularly singlet oxygen which then oxidizes the parent bisretinoid at carbon-carbon double bonds [10,11,26]. The oxygen-containing moieties that form within the photooxidized bisretinoid includes 3 membered rings that incorporated one oxygen atom (epoxide; C-O-C; epoxide-A2E), heterocyclic rings of 4 carbons and one oxygen (furan; furano-A2E), and heterocycles that include 4 carbons and an endoperoxide (O-O; peroxy-A2E). The endoperoxides readily undergo double bond lysis leading to the formation of aldehyde-bearing photofragments, including MG. Unlike free radicals, ketones and aldehydes are relatively long lived and therefore can diffuse from their site of origin to reach and attack other targets intra- or extracellularly. Thus, it is perhaps not surprising that we previously found that when A2E-containing RPE growing on fibronectin were irradiated to initiate A2E photodegradation, the fibronectin substrate became AGE modified [27].

**Figure 7:**
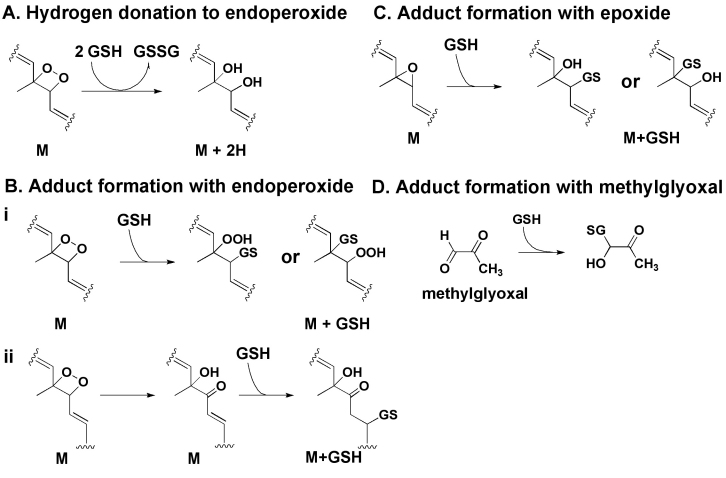
Proposed mechanisms for glutathione (GSH) interaction with photooxoA2E and methylglyoxal, an A2E photodegradation product. **A**: GSH would transfer two hydrogens from two GSH molecules to an endoperoxide on A2E, resulting in the *m/z*+2 pattern associated with photooxidized forms of A2E in the *m/z* 640–736 region of Figure 3B-D [33]. **B**: GSH adduct formation with an endoperoxide on A2E would occur via nucleophilic attack and ring opening [34,35]. Simple addition of GSH at the site of an endoperoxide would involve the formation of an unstable hydroperoxide (OOH) moiety (**B**, i) and for example would account for *m/z* 947 in Figure 4B, insert. Alternatively, GSH conjugation could involve attack of the endoperoxide bridge (O-O) by the GSH thiolate followed by carbonyl formation and GS insertion (**B**, ii); this mechanism would account for *m/z* 931 in Figure 4B, insert [33,36,37]. **C**: Adduct formation with an epoxide would be expected to occur [35]; however, the appropriate product (*m/z* 915) was not detected. **D**: GSH can react with methylglyoxal (MG) released upon A2E photodegradation to form an MG-GSH hemi-thioacetal [38]; this adduct accounts for *m/z* 380 and 402 in Figure 6F.

In the studies we are reporting here, we have demonstrated through a combination of colorimetric assays, chromatography, and MS that GSH can chemically reduce photooxidized A2E. GSH can also form adducts with both photooxidized A2E and photodegradation products of A2E. In Figure 7, we suggest possible routes by which GSH may react with photooxidized and photodegraded A2E. These findings complement our prior observation that sulforaphane, a phytochemical that increases the cellular content of GSH, can protect against the cellular damage associated with photooxidation of A2E [28]. In addressing specific A2E photocleavage products, we observed that GSH can form an adduct with MG, both noncatalytically and by GST mediation. Since MG damages proteins by reacting with amino and guanidine groups of lysine and arginine residues [29], the binding of GSH to these photocleavage products of A2E likely serves to limit their reactivity. GSH consumption was less pronounced when GSH was added to the mixture after irradiation, possibly because in the absence of GSH, A2E photofragments reacted among themselves.

While GSH is synthesized in the cell cytosol, degradation of GSH and GSH conjugates occurs only in the extracellular milieu. Thus, GSH-conjugate formation may be a mechanism for the elimination of electrophiles such as those generated by bisretinoid photooxidation/photodegradation. GSH conjugates can be exported from the cells by ATP-binding cassette transporters of the multi–drug resistance protein family or ral-binding guanosine triphosphate (GTP)ase activating protein 1 (RalBP1) [2,30], and once the GSH conjugate is released from the cells it is rapidly degraded by the plasma membrane–bound enzymes γ-glutamyl transpeptidase (γGT) and dipeptidases to release glutamate and glycine. The fate of the remaining cysteine S-conjugate is less clear. Once GSH has reacted with MG, the adduct can also be acted upon by the glyoxalase system, although the efficiency of this system decreases rapidly with a fall in GSH levels [31].

Besides reacting with GSH, thiol-reactive oxidation products of A2E could potentially react with essential thiols of critical proteins, resulting in the loss of protein function. This is an issue that we are currently investigating. Cellular levels of GSH can be diminished by inherited or acquired deficiencies in the enzyme transporters or transcription factors that are involved in GSH regulation. One form of macular degeneration, a rod/cone maculopathy [32], is attributable to GSH synthase deficiency and is inherited as an autosomal recessive disorder. The presence of macular edema and subnormal electrooculogram is suggestive of RPE cell involvement and may be consistent with the need for RPE to maintain generous levels of GSH, at least in part, to protect against the effects of bisretinoid photooxidation and cleavage.
